# Shade Avoidance 3 Mediates Crosstalk Between Shade and Nitrogen in Arabidopsis Leaf Development

**DOI:** 10.3389/fpls.2021.800913

**Published:** 2022-01-13

**Authors:** Xin-Yue Yang, Zhong-Wei Zhang, Yu-Fan Fu, Ling-Yang Feng, Meng-Xia Li, Qi Kang, Chang-Quan Wang, Ming Yuan, Yang-Er Chen, Qi Tao, Ting Lan, Xiao-Yan Tang, Guang-Deng Chen, Jian Zeng, Shu Yuan

**Affiliations:** ^1^College of Resources, Sichuan Agricultural University, Chengdu, China; ^2^College of Agronomy, Sichuan Agricultural University, Chengdu, China; ^3^Chengdu Botanical Garden, Chengdu, China; ^4^College of Life Science, Sichuan Agricultural University, Ya’an, China

**Keywords:** auxin accumulation, chlorophyll accumulation, leaf development, nitrogen level, shade

## Abstract

After nitrogen treatments, plant leaves become narrower and thicker, and the chlorophyll content increases. However, the molecular mechanisms behind these regulations remain unknown. Here, we found that the changes in leaf width and thickness were largely compromised in the *shade avoidance 3* (*sav3*) mutant. The *SAV3* gene encodes an amino-transferase in the auxin biosynthesis pathway. Thus, the crosstalk between shade and nitrogen in Arabidopsis leaf development was investigated. Both hypocotyl elongation and leaf expansion promoted by the shade treatment were reduced by the high-N treatment; high-N-induced leaf narrowing and thickening were reduced by the shade treatment; and all of these developmental changes were largely compromised in the *sav3* mutant. Shade treatment promoted *SAV3* expression, while high-N treatment repressed *SAV3* expression, which then increased or decreased auxin accumulation in cotyledons/leaves, respectively. SAV3 also regulates chlorophyll accumulation and nitrogen assimilation and thus may function as a master switch responsive to multiple environmental stimuli.

## Introduction

More greening but late maturity after the application of nitrogen fertilizer has been known for hundreds of years as an agricultural experience (Yuan et al., 2016), but the detailed molecular mechanisms of nitrogen-regulated leaf development and chlorophyll (Chl) accumulation are largely unknown. After nitrogen treatment, plant leaves become narrower and thicker, and the Chl content increases. In contrast, under a nitrogen-deficient condition, plant leaves become wider and thinner, and the Chl content decreases (Yuan et al., 2016).

The shade avoidance syndrome is a phenotype, including increased leaf angle, promoted hypocotyl elongation, and accelerated leaf senescence (Tao et al., 2008). SAS reduces deposition of fixed carbon to storage organs, resulting in re-allocation of energy resources from storage organs to stems and petioles so that the plant outgrows its competitors (Ballare, 1999). In response to prolonged shade, reproductive development is accelerated, potentially leading to decreased biomass, and seed yield (Franklin and Whitelam, 2005). Nevertheless, effects of shade on leaf width and thickness and possible crosstalk between shade and nitrogen in leaf development have not been documented before.

Auxin is an essential plant hormone that regulates cell growth and division, organogenesis, and the responses of plants to various external stimuli (Yan et al., 2017). Auxin is a highly concentration-dependent hormone in plants that regulates all of the major processes of plant development. It has been reported that a dynamic auxin concentration gradient is established across plant organs (Tao et al., 2008; Zhao, 2018). Furthermore, the local auxin level adjusts quickly in response to environmental changes (Tao et al., 2008; Zhao, 2018). Polar auxin transport plays a key role to modulate local auxin concentrations (Friml, 2003; Blilou et al., 2005), but local auxin generation determines the overall auxin level within a plant and also plays an important role in plant development and the adaptation to environmental changes (Tao et al., 2008; Zhao, 2018).

In this study, 15 auxin-related and nitrogen-signal-related Arabidopsis mutants were used to identify the key factors mediating nitrogen-regulated leaf development. Interestingly, changes in leaf width and thickness were compromised only in the *shade avoidance 3* (*sav3*) mutant. The *SAV3* gene encodes an amino-transferase and catalyzes the formation of indole-3-pyruvic acid (IPA) from L-Trp, which is the first step in the IPA-dependent indoleacetic acid (IAA) biosynthetic pathway (Stepanova et al., 2008; Tao et al., 2008; Zhao, 2010). The *sav3* mutant displays defects in response to vegetative shade, gravity, and hormones including ethylene and cytokinin, indicating that SAV3-mediated auxin biosynthesis is an essential component in these responses (Stepanova et al., 2008; Tao et al., 2008; Zhou et al., 2011; Yang et al., 2014; Yan et al., 2017). Importantly, shade avoidance responses (hypocotyl elongation and leaf expansion) were largely compromised in the *sav3* mutant (Tao et al., 2008).

Therefore, there should be crosstalk between shade and nitrogen in Arabidopsis leaf development, both of which are mediated by SAV3. In this study, we found that both hypocotyl elongation and leaf expansion promoted by the shade treatment were reduced by the high-N treatment; high-N-induced leaf narrowing and thickening were reduced by the shade treatment; and all of these developmental changes were largely compromised in the *sav3* mutant. SAV3 also regulates Chl accumulation and nitrogen assimilation, thus functioning as a master switch responsive to multiple environmental stimuli.

## Materials and Methods

### Material and Plant Growth Conditions

The mutants used in this study were all constructed in the background of Columbia wild-type *Arabidopsis thaliana*. The *sav3-1*, *sav3-2*, *35S::SAV3,sav3-2*, *DR5::GUS*,Col-0, and *SAV3p::SAV3-GUS* were obtained from Prof. Yi Tao (Xiamen University, China). *DR5::GUS,sav3-2* was acquired by performing genetic crossing and the progenies were analyzed by PCR. *afb3* (Auxin Signaling F-Box 3; SALK_016356C), *arf8* (Auxin Response Factor 8; CS24608), *cry1* (Cryptochrome 1; CS6955), *cry2* (Cryptochrome 2; CS3732), *phyB* (Phytochrome B; SALK_022035), *cyp79b2* (Cytochrome P79B2; SALK_130570C), *cyp79b3* (Cytochrome P79B3; CS311371), *nlp7-1* (NIN Like Protein 7; SALK_026134C), *pils-7* (PIN-LIKES 7; SALK_068682C), *tir1-1* (Transport Inhibitor Response 1; CS3798), *tir4* (Transport Inhibitor Response 4; SALK_201946C) mutants, *nia1,2* (Nitrate Reductase 1 and 2; CS2356) and *tar1,2* (Tryptophan Aminotransferase Related 1 and 2; CS16410) double mutants, and *pif3,4,5* (Phytochrome-Associated Protein 3, 4 and 5; CS66048) triple mutant were obtained from the Arabidopsis Biological Resource Center (ABRC) at Ohio State University (Columbus, OH, United States) and were all in the Col-0 background. After vernalization at 4°C for 3 days, the seeds were sterilized in 75% (v/v) alcohol and 0.1% (v/v) HgCl_2_, and then grown on 1/2 MS medium with 1% sucrose containing different concentrations of nitrogen. According to our previous study (Yuan et al., 2016), three different concentrations of nitrogen were set up: low-nitrogen (1/20 N; LN), normal-nitrogen (1/2 N; NN), high-nitrogen (2 N; HN). MS media contain 20 mM NH_4_NO_3_ and 18.8 mM KNO_3_ (58.8 mM nitrogen). For 1/20 N growth condition, NH_4_NO_3_ and KNO_3_ were reduced to 1 and 0.94 mM, respectively, and complemented with 19 mM NaCl and 17.86 mM KCl. For 2 N growth condition, NH_4_NO_3_ and KNO_3_ were enhanced to 40 and 37.6 mM, respectively.

The temperature and humidity were 23°C and 70%. The light intensity under continuous white light (Wc) condition was 100 μmol⋅m^–2^⋅s^–1^, and shade condition was 15 μmol⋅m^–2^⋅s^–1^. All plant materials were grown under Wc condition for 2 days for germination, then grown under Wc condition and shade condition respectively. A part of 5-day-old wild-type seedlings under Wc were subjected to 8-h shade treatment. A part of 14-day-old wild-type seedlings under Wc were subjected to 8-h shade treatment.

### Determination of Blade Width and Thickness

All the materials were grown for 14 days under Wc condition or shade condition with low, normal or high N levels, and the plants with the same growth trend were selected. The samples were taken at the position where the leaf of the first true leaf was the widest and perpendicular to the vein. The paraffin sections were made by staining with safranine and solid green. The slices were scanned and imaged under the stereo microscope (Leica Microsystems M165C). The collected images were analyzed by the ImageJ (Schneider et al., 2012), and the width and average thickness of the blade were measured according to the scale length of the collected images. A total of 15 seedlings were used for each material under each treatment.

### Determination of Hypocotyl Length and Leaf Angle

The hypocotyl length and leaf angle of 5-day-old Arabidopsis grown under Wc condition or shade condition with low, normal or high N levels were measured by the ImageJ (Schneider et al., 2012). A total of 15 seedlings were used for each material under each treatment. The experimental methods were set as described previously (Tao et al., 2008).

### GUS Staining

Five-day-old and 14-day-old Arabidopsis grown under Wc condition or shade condition with low, normal or high N levels were used for GUS staining by GUS Staining Kit (Solarbio Comp., Beijing, China). Briefly, histochemical localization of GUS was carried out in a solution containing 5 mg mL^–1^ 5-bromo-4-chloro-3-indolyl-β-D-glucuronide as the substrate in a buffer containing 100 mM phosphate buffer (pH 7.0) and incubating at 37°C. After staining, tissue was incubated in 70% (v/v) ethanol to remove chlorophyll and reduce background.

### Germination Rate Determination

The germination rate under low, normal or high N levels and different concentrations of GA_3_ (0, 10, 25, and 100 μM) or IAA (0, 1, and 10 μM) was determined. GA and IAA were added to vernalization seeds and medium. The experimental methods were set as described previously (Luo et al., 2021).

### Determination of Fresh Weight and Dry Weight

Seven-day-old, 14-day-old, 21-day-old, and 28-day-old Arabidopsis grown under low, normal or high N levels with or without shade treatment were used for fresh weight and dry weight determination. The experimental methods were set as described previously (Zhang et al., 2020a).

### Determination of Chlorophyll Contents

Fourteen-day-old Arabidopsis grown under Wc condition or shade condition with low, normal or high N levels were used for chlorophyll contents determination. Cut about 0.5 g leaves into pieces and put them in a mortar. Added 80% (v/v) acetone, ground and filtered in a 25 mL volumetric flask. Rinsed the mortar repeatedly with 80% (v/v) acetone and constant volume to 25 mL. Draw 1 mL of extract and diluted to 10 mL. With 80% (v/v) acetone as reference, the absorbance values were measured at 645 and 663 nm. The calculation methods of chlorophyll contents were set as described previously (Lichtenthaler and Wellburn, 1983).

ImageJ was used to measure the leaf area, and the chlorophyll contents per unit area was calculated according to the chlorophyll contents.

### Determination of Nitrogen Contents

Fourteen-day-old Arabidopsis grown under Wc condition or shade condition with low, normal or high N levels were used for nitrogen content determination. For total nitrogen determination, the dried sample was crushed through 100 mesh sieve and dried to constant weight at 55°C, and then analyzed with an element analyzer (Elementar vario MACRO cube, Langenselbold, Germany).

Whole seedling nitrate and ammonium contents were determined as described in Orsel et al. (2004). The dried samples were digested with H_2_SO_4_ and then detected with a flow injection analysis equipment (FIAstar 5000 analyser; FOSS, Hilleroed, Denmark) (Chen et al., 2018).

### Determination of Nitrate Reductase Activity

The NR activity was measured according to the methods of Scheible et al. (1997) and Zhao et al. (2009) with some modifications. About 1 g of leaves was ground with liquid N_2_ and then resuspended in extraction buffer with 10% glycerol, 1% polyvinylpyrrolidone, 0.5 mM phenylmethylsulfonyl fluoride, and 1 mM leupeptin (pH 7.5). The enzyme activity was determined adding 5 volumes of assay buffer (0.25 mM NADH, 5 mM KNO_3_, 100 mM HEPES-KOH, and pH 7.5). The nitrite products were measured colorimetrically at 520 nm by adding 1% sulfanilamide in 3 M HCl and 0.02% N-(1-naphthyl)-ethylenediamine.

### Quantitative Real-Time PCR

Five-day-old *DR5::GUS Col-0* and *DR5::GUS sav3-2* grown under Wc condition or shade condition with low, normal or high N levels were used for RNA extraction with the TRIzol™ Plus RNA Purification Kit (Invitrogen, Carlsbad, CA, United States). All RNA samples were treated with DNase I before RT-PCR. For each sample, 1 μg RNA was subjected to cDNA synthesis by using SYBR Premix Ex Taq (Takara Biotechnology Dalian Co., Ltd., Dalian, China). Then the quantitative PCR (qPCR) was performed with the EmeraldAmp MAX PCR Master Mix (Takara Biotechnology). The threshold cycle (Ct), defined as the PCR cycles when the product could be first detected, was measured to the determine relative expression levels of target gene *SAV3* (forward primer: CTCGAGGAAACCCGAAAAAAT; reverse primer: CTGGCTCAAGGAACCAACACA). Three biological replicates with three technical repetitions were performed for each sample. *ACTIN7* gene was used as an internal control (forward primer: ATCCCTCAGCACCTTCCAAC; reverse primer: ACCCGATACTTAAATAATTGTCTCAT). Normalization of qPCR data was achieved by subtracting the Ct value of the internal reference gene from the Ct values of the target genes to get ΔCt. The individual gene expression levels were presented as the fold-changes relative to *ACTIN7* expression levels (2^–Δ*Ct*^). The expression level of the seedlings grown under continuous white light (Wc) with normal N level (NN) was normalized into “1.0” (Zhang et al., 2020b).

### Quantification of Free Indoleacetic Acid

Five-day-old seedlings grown under Wc condition or shade condition with low, normal, or high N levels were used for quantification of free IAA. Seedlings were pooled in triplicates, weighed, and frozen in liquid nitrogen for quantification of free IAA content. The frozen sample containing about 50 mg of tissue (fresh weight) was homogenized in 0.5 mL of 50 mM sodium phosphate buffer, pH 7.0, containing 0.02% diethyldithiocarbamic acid (antioxidant) and 500 pg ^13^C_6_-IAA internal standard (Beijing Yaolai Biology Comp., Beijing, China), using the Retsch vibration mill (Retsch) and a 3-mm tungsten carbide bead at a frequency of 30 Hz for 2 min. The sample was then incubated for 15 min at 4°C with continuous shaking. The pH was adjusted to 2.7 with 1 M HCl, and the sample was purified by solid phase extraction on a 500-mg Isolute C8-EC column conditioned with 2 mL of methanol and 2 mL of 1% acetic acid. The column was washed with 2 mL of 10% methanol in 1% acetic acid and eluted with 2 mL 70% methanol in 1% acetic acid, and the sample was evaporated to dryness. The sample was dissolved in 0.2 mL of 2-propanol and 1 mL of dichloromethane, and IAA was methylated by adding 5 μL 2 M trimethylsilyl-diazomethane in hexane and incubating the sample at room temperature for 30 min. Five microliters of 2 M acetic acid in hexane was added to destroy excess diazomethane, and the sample was then evaporated to dryness. The methylated sample was then trimethyl-silylated and analyzed by gas chromatography–selected reaction monitoring–mass spectrometry as described previously (Andersen et al., 2008).

### Statistical Analysis

Leaf width and thickness, hypocotyl length, leaf angle, leaf area, and the chlorophyll contents per unit area were measured with ImageJ software. The data for qRT-PCR were collected with Bio-Rad real-time PCR detection systems with gene-specific primers. For recording of hypocotyl length, leaf angles, leaf width, leaf thickness, leaf area, and plant growth, 15 seedlings were used for each material under each treatment. For the other experiments, at least 3 independent replicates were performed. The data in figures were statistically analyzed using two-way ANOVA with the SPSS 22.0 software (IBM Comp., Chicago, IL, United States). The Duncan’s multiple range test was performed to compare the means. The different letters were considered to be statistically significant at *P* < 0.05.

## Results

### Identification of Arabidopsis Mutants Involved in N-Regulated Leaf Development

**FIGURE 1:**
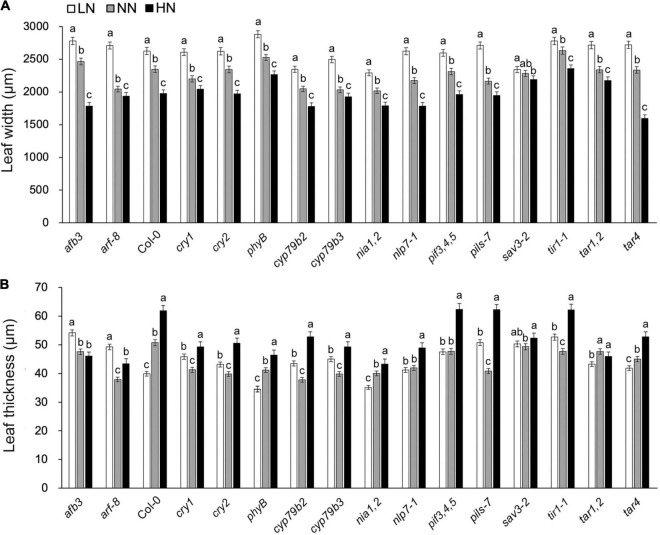
Changes in leaf width/thickness under different N levels were compromised in *sav3* mutant. A total of 15 auxin-related or nitrogen-signal-related Arabidopsis mutants were enrolled. Maximum widths **(A)** and average thicknesses **(B)** of 14-day-old leaves grown under different N levels were determined. LN, low N condition; NN, normal N condition; HN, high N condition. Bars represent standard deviations of 15 independent replicates. Values of each plant material followed by different letters are significantly different at *P* < 0.05 according to Duncan’s multiple range test.

To identify Arabidopsis genes that are involved in N-regulated leaf development, we performed a reverse genetic screen for leaves that did not become narrower and thicker after the high-N treatment. A total of 15 auxin-biosynthesis or auxin-signaling related Arabidopsis mutants were enrolled, including the blue-light receptor cryptochrome 1 (CRY1), functioning in nitrogen regulation of flowering (Yuan et al., 2016) and nitrate reductase (NIA), a key enzyme responsible for nitrogen-regulated auxin accumulation in Arabidopsis roots (Fu et al., 2020). Both leaf width and leaf thickness decreased as the nitrogen level increased. However, they were almost unchanged in the *sav3* mutant under different N levels (Figures 1A,B). In summary, SAV3 may be responsible for N-regulated leaf development.

During the above experiments, we found an interesting phenomenon that germination rates were significantly reduced under the high-N condition in *sav3* mutants (<20%), but not in the wild-type seeds (Supplementary Figure 1). Considering that SAV3 is a key enzyme in the IAA biosynthetic pathway, the seeds were treated with different concentrations of IAA (0, 1, and 10 μM) during germination under light. Two *sav3* mutants, a weak mutant *sav3-1* (with less-changed hypocotyl length and leaf angle under shade) and a mull mutant *sav3-2* (with almost unchanged hypocotyl length and leaf angle under shade), and genetic complementation material *35S::SAV3, sav3-2* were investigated (Tao et al., 2008). Differences in germination rates among *sav3-1*, *sav3-2*, *35S::SAV3, sav3-2*, and wild-type plants were eliminated by exogenous IAA treatments (Supplementary Figure 1), implying a role of IAA in germination under the high-N condition. Similar to IAA, moderate levels of exogenous gibberellin treatments (10 or 25 μM GA_3_) also promoted the germination of all plants; however, the germination rates of the *sav3-1* and *sav3-2* mutants were still significantly lower than those of the *35S::SAV3, sav3-2*, and wild-type plants (Supplementary Figure 1), implying that GA may be not involved in the germination repression in *sav3* mutants under high-N conditions.

### Crosstalk Between Shade and Nitrogen in Hypocotyl Elongation and Leaf Expansion

**FIGURE 2:**
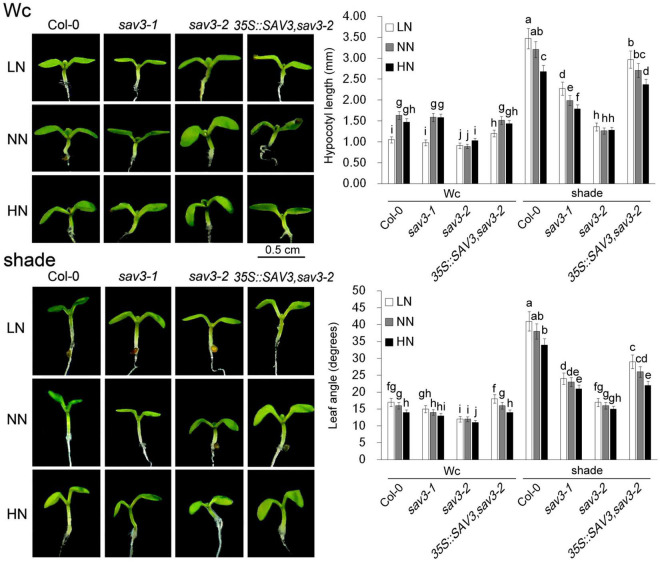
Shade avoidance responses were reduced by high-N treatment. Hypocotyl lengths and leaf angles of 5-day-old seedlings under different N levels with or without shade treatment were recorded. Wc, continuous white light; LN, low N condition; NN, normal N condition; HN, high N condition. Phenotypes are shown in the left panel; quantitative data are shown in the right panel. Bars represent standard deviations of 15 independent replicates. Values followed by different letters are significantly different at *P* < 0.05 according to Duncan’s multiple range test.

It is well-known that SAV3 is an IAA biosynthetic enzyme, which can be exponentially induced by shade (Stepanova et al., 2008; Tao et al., 2008; Zhao, 2010). Thus, crosstalk between shade and nitrogen in shade avoidance responses was investigated; the hypocotyl lengths and leaf angles of 5-day-old seedlings under different N levels with or without shade treatment were recorded. Hypocotyl length doubled under the shade condition but declined under the high-N condition, with the exception that seedlings under continuous white light (Wc) and low-N had the shortest hypocotyls (Figure 2). The leaf angle had a similar pattern, i.e., it increased exponentially under the shade condition but declined under the high-N condition (Figure 2). The response of the *sav3* mutants to both shade treatment and different levels of N was weaker than that of the wild-type seedlings. The *sav3-2* mutant had the shortest hypocotyls with the smallest leaf angles in all of the cases (Figure 2).

**FIGURE 3:**
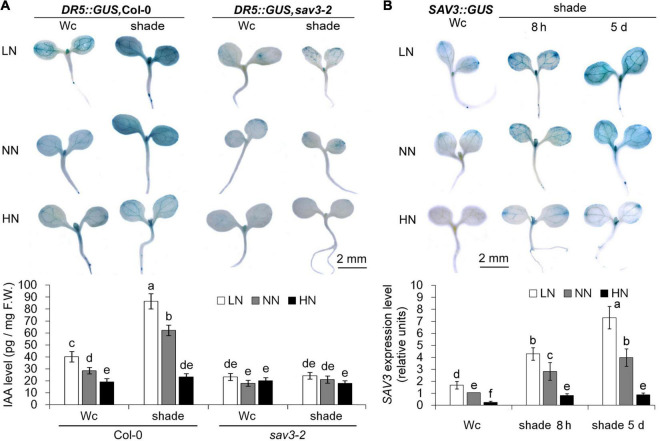
Shade avoidance 3-mediated auxin accumulation under shade conditions was reduced by high-N treatment. **(A)** Auxin accumulation pattern in 5-day-old wild-type (Col-0) and *sav3-2* mutant seedlings under different N levels with or without shade treatment. The levels of auxin were reflected by DR5::GUS expression. Quantitative data of free IAA are shown in the lower panel. F.W., fresh weight; Wc, continuous white light; LN, low N condition; NN, normal N condition; HN, high N condition. **(B)** *SAV3* expression pattern in 5-day-old wild-type seedlings under different N levels with or without (8-h or 5-day) shade treatment. The levels of *SAV3* gene expression were reflected by SAV3::GUS expression. Quantitative real-time PCR data of SAV3 transcript are shown in the lower panel. The individual gene expression levels were presented as the fold-changes relative to *ACTIN7* expression levels. The expression level of the seedlings grown under continuous white light (Wc) with the normal N level (NN) was normalized into “1.0.” Bars represent standard deviations of three independent replicates. Values followed by different letters are significantly different at *P* < 0.05 according to Duncan’s multiple range test.

To investigate the site of auxin accumulation under different N levels and following shade treatment, we used transgenic plants harboring a DR5::GUS reporter, an artificial auxin reporter gene construct, whose expression indirectly reflects the levels of free auxin (Sabatini et al., 1999; Aloni et al., 2003; Mallory et al., 2005; Cheng et al., 2006). DR5::GUS expression levels increased in cotyledons and the other aerial tissues after either 8 h or 5 days of shade treatment but decreased significantly in all aerial tissues (especially vascular tissues) under the high-N condition (Figure 3A). By contrast, low expression of DR5::GUS in cotyledons and no expression of DR5::GUS in hypocotyls of 5-day-old *sav3-2* seedlings were observed (Figure 3A). Of interest, the expression pattern of SAV3 in the shoot was similar to that of DR5::GUS (Figure 3B). All of these GUS staining data are consistent with the phenotype changes in shade avoidance responses.

### Crosstalk Between Shade and Nitrogen in Leaf Widening and Thickening

**FIGURE 4:**
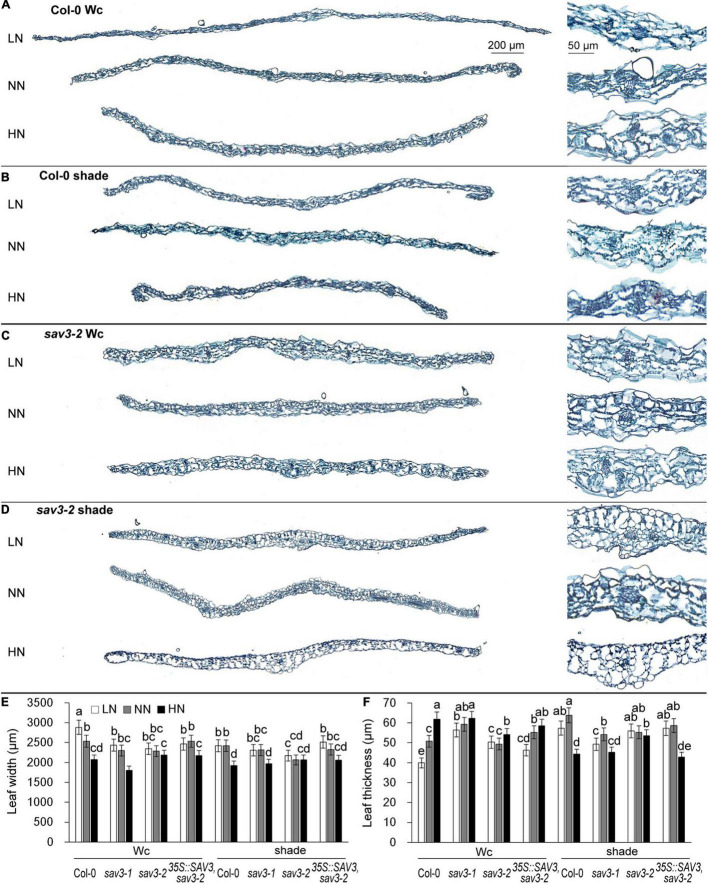
High-N-induced leaf narrowing and thickening were reduced by shade treatment. Cross sections of 14-day-old wild-type (Col-0) and *sav3-2* mutant leaves grown under different N levels and continuous white light (Wc) **(A,C)** or shade condition **(B,D)**. Local enlarged images of the leaf central vein are shown in the corresponding right panels. LN, low N condition; NN, normal N condition; HN, high N condition. **(E)** Maximum widths of 14-day-old wild-type (Col-0), *sav3-1*, *sav3-2*, and *35S::SAV3,sav3-2* leaves. **(F)** Average thicknesses of 14-day-old wild-type (Col-0), *sav3-1*, *sav3-2*, and *35S::SAV3,sav3-2* leaves. Bars represent standard deviations of 15 independent replicates. Values followed by different letters are significantly different at *P* < 0.05 according to Duncan’s multiple range test.

Crosstalk between shade and nitrogen in true leaf development was investigated: leaf widths and thicknesses of 14-day-old seedlings under different N levels with or without shade treatment were recorded. Leaf width significantly declined but leaf thickness significantly increased under the high-N condition (Figures 4A,E,F). The shade treatment reduced the extent of these changes, with the exception that seedlings under the shade condition and high-N had the thinnest leaves (Figures 4B,E,F), possibly because of an extremely low C/N ratio. The response of the *sav3* mutants to both shade treatment and different levels of N was weaker than that of the wild-type seedlings (Figures 4C–F and Supplementary Figures 2A–D). The leaf width and thickness of the *sav3-2* mutant were almost unchanged in all cases (Figures 4C–F). Given that leaves become narrower and thicker after nitrogen treatment, the ratio of leaf area to fresh weight decreased more dramatically under the high-N condition in *35S::SAV3, sav3-2*, and wild-type plants but did not decline in the *sav3-2* mutant (Supplementary Figure 3).

**FIGURE 5:**
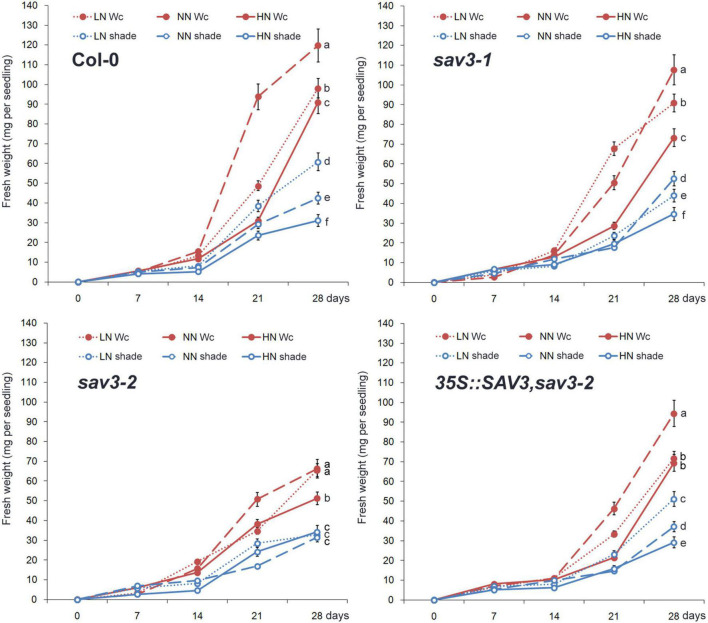
Plants grew fastest under normal N condition and continuous white light. Fresh weight increasing of wild-type (Col-0), *sav3-1*, *sav3-2*, and *35S::SAV3,sav3-2* seedlings grown under different N levels with or without shade treatment were recorded every week. Wc, continuous white light; LN, low N condition; NN, normal N condition; HN, high N condition. Bars represent standard deviations of 15 independent replicates. Values at 28th day followed by different letters are significantly different at *P* < 0.05 according to Duncan’s multiple range test.

Plant growth was assessed by recording the fresh weight every week. The highest growth rate occurred after the normal N treatment under continuous white light (Wc) (Figure 5), because that either low or high N treatment provides a stress to the seedlings. Plant growth was strongly inhibited under the shade condition (decreased to about 1/3 of that under Wc). Interestingly, under the shade condition, the highest growth rate occurred after the low N treatment (Figure 5), may because that shade treatment greatly declined the C:N ratio. The response of the growth of the *sav3-2* mutant to both shade treatment and different levels of N was largely compromised (Figure 5).

Auxin accumulation and the expression pattern of SAV3 in 14-day-old seedlings were similar to those of 5-day-old seedlings in that the expression increased after shade treatment but decreased under the high-N condition. Nevertheless, much higher expression of DR5::GUS in 14-day-old *sav3-2* seedlings was observed compared with the 5-day-old *sav3-2* seedlings (Figure 2), suggesting that auxin accumulation was not completely abolished in the mutant.

### Shade Avoidance 3 Mediates Chlorophyll Accumulation and Nitrogen Assimilation

**FIGURE 6:**
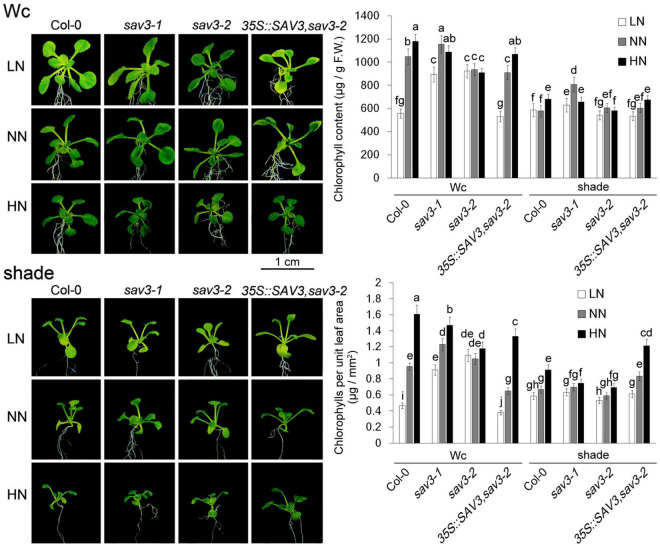
Chlorophyll accumulation induced by high-N treatment declined under shade condition. Chl content per fresh weight and Chl content per unit leaf area of 14-day-old seedlings under different N levels with or without shade treatment were determined. F.W., fresh weight; Wc, continuous white light; LN, low N condition; NN, normal N condition; HN, high N condition. Phenotypes are shown in the left panel; quantitative data are shown in the right panel. Bars represent standard deviations of three independent replicates. Values followed by different letters are significantly different at *P* < 0.05 according to Duncan’s multiple range test.

The Chl content per fresh weight of leaves decreased under the low-N treatment but increased in the high-N treatment. Given that leaves become wider and thinner under low-N conditions while leaves become narrower and thicker under high-N conditions, more dramatic changes in the Chl content per unit leaf area were observed (Figure 6). Thus, more greening of leaves after nitrogen treatment could be attributed to both the morphological changes of the leaves and enhanced Chl biosynthesis. Shade treatment reduced Chl contents as well as the changes in Chl contents induced by different levels of nitrogen (Figure 6). In other words, shade treatment over-ruled the accumulation of Chl caused by the N increase. Again, the Chl accumulation in the *sav3-2* mutant in response to different levels of N was largely compromised. The Chl content per fresh weight of leaves in the *sav3-2* mutant was unchanged under different N levels (Figure 6), indicating that SAV3 mediates N-regulated Chl biosynthesis.

**FIGURE 7:**
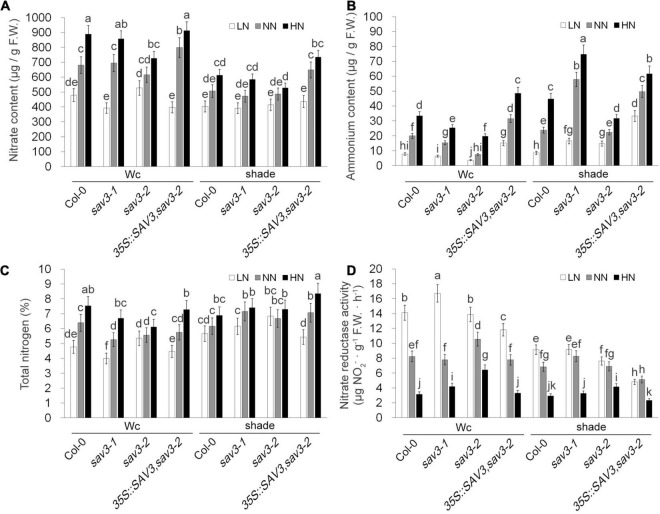
Shade avoidance 3 mediates N-regulated and shade-regulated nitrogen assimilation. Nitrate content **(A)**, ammonium content **(B)**, total N content **(C)** and nitrate reductase activity **(D)** of 14-day-old seedlings grown under different N levels with or without shade treatment were determined. F.W., fresh weight; Wc, continuous white light; LN, low N condition; NN, normal N condition; HN, high N condition. Bars represent standard deviations of three independent replicates. Values followed by different letters are significantly different at *P* < 0.05 according to Duncan’s multiple range test.

The nitrate content, ammonium content, and total N content increased in the high-N treatment (Figures 7A–C), whereas nitrate reductase activity decreased in this treatment (Figure 7D). Shade treatment reduced all these contents as well as the changes in these contents induced by different levels of nitrogen (Figures 7A–D). Interestingly, the extent of these changes was largely reduced in the *sav3-2* mutant (Figures 7A–D), suggesting that SAV3 may also mediate nitrogen assimilation processes.

## Discussion

This is the first report on the crosstalk between shade and nitrogen in Arabidopsis leaf development. In general, shade treatment mainly promoted hypocotyl elongation and cotyledon expansion, which could be partly reduced by the high-N treatment, while nitrogen treatment mainly induced leaf narrowing and thickening, which could be partly reversed by the shade treatment.

Plants sense and respond to light through multiple photoreceptors including phytochromes (Goyal et al., 2016) and cryptochromes (Pedmale et al., 2016). The decreased ratio of red to far-red light or low blue light that occurs under a canopy triggers the shade-avoidance response (Pedmale et al., 2016). The leaves (cotyledons) act as a photoperceptive organ in this response. Downstream of photoperception, SAV3 and the YUCCA family of flavin monooxygenases promote auxin biosynthesis in cotyledons and hypocotyls (Tao et al., 2008; Goyal et al., 2016). Then, through polar auxin transport, auxin gradients are established, which subsequently direct the shade-avoidance response. A protein involved in the polar auxin transport has recently been identified: Shade Avoidance 4 (SAV4) (Ge et al., 2017). Membrane-localized SAV4 protein shows a polar association with the shoot-ward plasma membrane domain in hypocotyl cells, which may be necessary for hypocotyl elongation. Polarly localized SAV4 inhibits auxin efflux toward shoots and facilitates the establishment of proper auxin gradients under the shade condition (Ge et al., 2017). Further study by Kim et al. (2018) suggested that the shade stimulus is spatially processed within the cotyledon. They found that more genes were upregulated by shade treatment in vascular tissues than in epidermal and mesophyll tissues. These genes mainly included auxin-responsive genes, suggesting that auxin acts as an important intra-organ signaling molecule that controls the vascular shade responses within the cotyledon (Kim et al., 2018). Consistent with this report, in this study, we found that *SAV3* gene expression and auxin accumulation occurred mainly in the vascular tissues of cotyledons and hypocotyls.

The interactions between nitrogen nutrition and auxin signaling have also been well documented (Rubio et al., 2009; Krouk et al., 2011). In general, N deficiency dramatically reduced auxin accumulation in shoot tips and leaves. However, low nitrate levels may also increase the root auxin content (Caba et al., 2000; Walch-Liu et al., 2006; Tian et al., 2008) and enhance the shoot-to-root transport of IAA, which results in higher IAA accumulation in roots (Tian et al., 2008). The genes that mediate auxin influx [such as *AUX1* (auxin transporter protein 1), *LAX2* (AUX1-like permease 2), and *LAX3*] and efflux [such as *PIN1* (PIN-FORMED 1), *PIN2*, *PIN4*, and *PIN7*] have been suggested to be regulated transcriptionally by N levels or the C/N ratio (Gutiérrez et al., 2007; Li et al., 2011). In addition to IAA carriers, nitrate transporters may also regulate the auxin accumulation pattern (Malamy, 2005; Krouk et al., 2010). For instance, the nitrate transporter NRT1.1 functions as a key auxin transporter and determines auxin levels in lateral root tips in response to N supply; a mutation in this transporter would alter the response of lateral root density to N availability (Krouk et al., 2010). NRT1.1 regulates nitrate-mediated auxin biosynthesis and signaling directly. AUX1 and PIN2 mediate auxin transports and function epistatic to NRT1.1 in low-nitrate-induced root architecture alternations (Chai et al., 2020). NRT1.1-induced repression of TAR2 (tryptophan aminotransferase related 2), and LAX3 is also involved in N-mediated lateral root development (Maghiaoui et al., 2020). Moreover, TAR2 was expressed in the pericycle and vascular tissues of roots and could be upregulated by low-nitrate treatment. A mutation in TAR2 resulted in almost no lateral roots in response to low nitrate (Ma et al., 2014). A recent study indicated that ammonium uptake may acidify the root apoplasts, which increase the pH-dependent influx of protonated auxin into epidermal and cortical cells and then stimulate lateral root emergence, while in N-deficient seedlings, auxin also accumulates in the root vascular tissues, but more alkaline apoplasts lead to the retention of auxin in these tissues and prevent lateral root emergence (Meier et al., 2020). Thus far, however, most previous studies focused on the N-mediated auxin accumulation pattern in root development. There are few reports on the role of auxin-related genes in leaf (or shoot) development. Here, we add new information that SAV3, an amino-transferase for IAA biosynthesis, may be involved in leaf narrowing and thickening under high-N conditions.

In addition to hypocotyl elongation and leaf development, SAV3 also mediates chlorophyll accumulation and nitrogen assimilation. The importance of auxin in the induction of chlorophyll has been reported in both algae and plants (Salazar-Iribe and De-la-Peña, 2020). Recently, it was reported that in *Camellia sinensis* seedlings exposed to shade and with a high accumulation of chlorophyll, a positive correlation between auxin and genes involved in chlorophyll biosynthesis such as HEMA1 (glutamyl-tRNA reductase 1), PORA (protochlorophyllide oxidoreductase A), CLH1 (chlorophyllase 1), and CAO (chlorophyllide a oxygenase) was observed (Liu et al., 2020). Tomato auxin response factor 10 (ARF10) positively regulates the expression of GLK1 (Golden2-Like 1), POR, CBP1 (chlorophyll binding protein 1), and CBP2, which are correlated with chlorophyll metabolism (Yuan et al., 2018). SAV3 might regulate chlorophyll biosynthesis indirectly by altering the auxin accumulation pattern and/or auxin signaling.

There are few reports about how auxin regulates nitrogen assimilation in plants. The rice *pin1b* mutant is hypersensitive to N starvation and altered the induction of several N-assimilation genes under low-N conditions (Gho et al., 2021). Our previous report demonstrated that the root auxin level was positively correlated with nitrate reductase activity (Fu et al., 2020). Indeed, CHLORATE1 (CHL1/NRT1.1) is thought to be responsible for the basipetal flux of auxin from the lateral root primordium under low-N conditions (discussed above) (Krouk et al., 2010; Chai et al., 2020; Maghiaoui et al., 2020). Crosstalk among SAV3, NRT1.1 and nitrate reductase needs further investigation.

In general, shade and nitrogen counteract each other in leaf development. In agricultural industry, shading often occurs in intercropping systems. By regulating the nitrogen level, the plant architectural traits may be changed to better adapt to the shade condition. And a key gene, *SAV3*, involved in crosstalk between shade and nitrogen has been identified, which may provide a useful reference for breeding of N-efficient crop cultivars and shade-tolerant crop cultivars. Moreover, we found that SAV3 regulates chlorophyll biosynthesis through changing the auxin level and/or accumulation pattern. Whether auxin regulates chlorophyll metabolism genes or enzymes directly at the transcriptional level and/or the translational level needs further investigations.

## Data Availability Statement

The original contributions presented in the study are included in the article/Supplementary Material, further inquiries can be directed to the corresponding author.

## Author Contributions

Z-WZ and SY conceived the project. SY designed the study. X-YY, Z-WZ, Y-FF, L-YF, M-XL, and QK performed the experiments. C-QW, MY, Y-EC, QT, TL, X-YT, G-DC, and JZ analyzed the data. X-YY and SY wrote the manuscript with input from X-YY, Z-WZ, and Y-FF. All authors contributed to the article and approved the submitted version.

## Conflict of Interest

The authors declare that the research was conducted in the absence of any commercial or financial relationships that could be construed as a potential conflict of interest.

## Publisher’s Note

All claims expressed in this article are solely those of the authors and do not necessarily represent those of their affiliated organizations, or those of the publisher, the editors and the reviewers. Any product that may be evaluated in this article, or claim that may be made by its manufacturer, is not guaranteed or endorsed by the publisher.
