# Supplementary material for: Shade Avoidance 3 Mediates Crosstalk Between Shade and Nitrogen in Arabidopsis Leaf Development
Source: Front Plant Sci. 2022 Jan 13;12:800913. doi: 10.3389/fpls.2021.800913 (PMC8792756; doi:10.3389/fpls.2021.800913)
Supplement: Supplementary file 1 [file Data_Sheet_1.PDF]

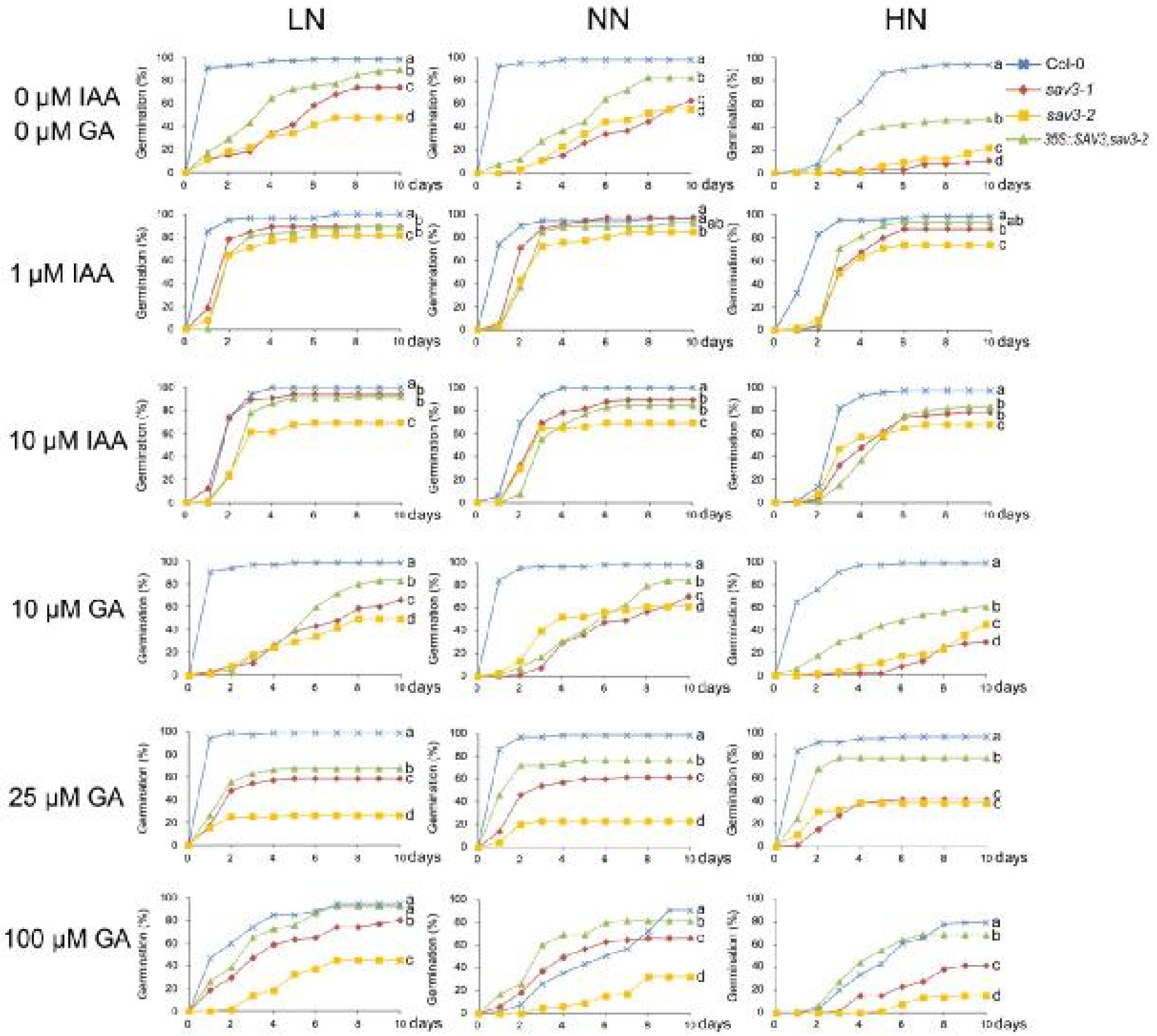

FIGURE S1 | Germination was significantly inhibited by high-N treatment in *sav3* mutants. Time-course germination of wild-type (Col-0), *sav3-1*, *sav3-2* and *35S::SAV3,sav3-2* seeds under different N levels and different concentrations of GA_3 (0 μM, 10 μM, 25 μM, 100μM) or IAA (0 μM, 1 μM, 10 μM) was determined. LN, low N condition; NN, normal N condition; HN, high N condition. Bars represent standard deviations of 3 independent replicates. Values at 10th day followed by different letters are significantly different at $P < 0.05$ according to Duncan's multiple range test.

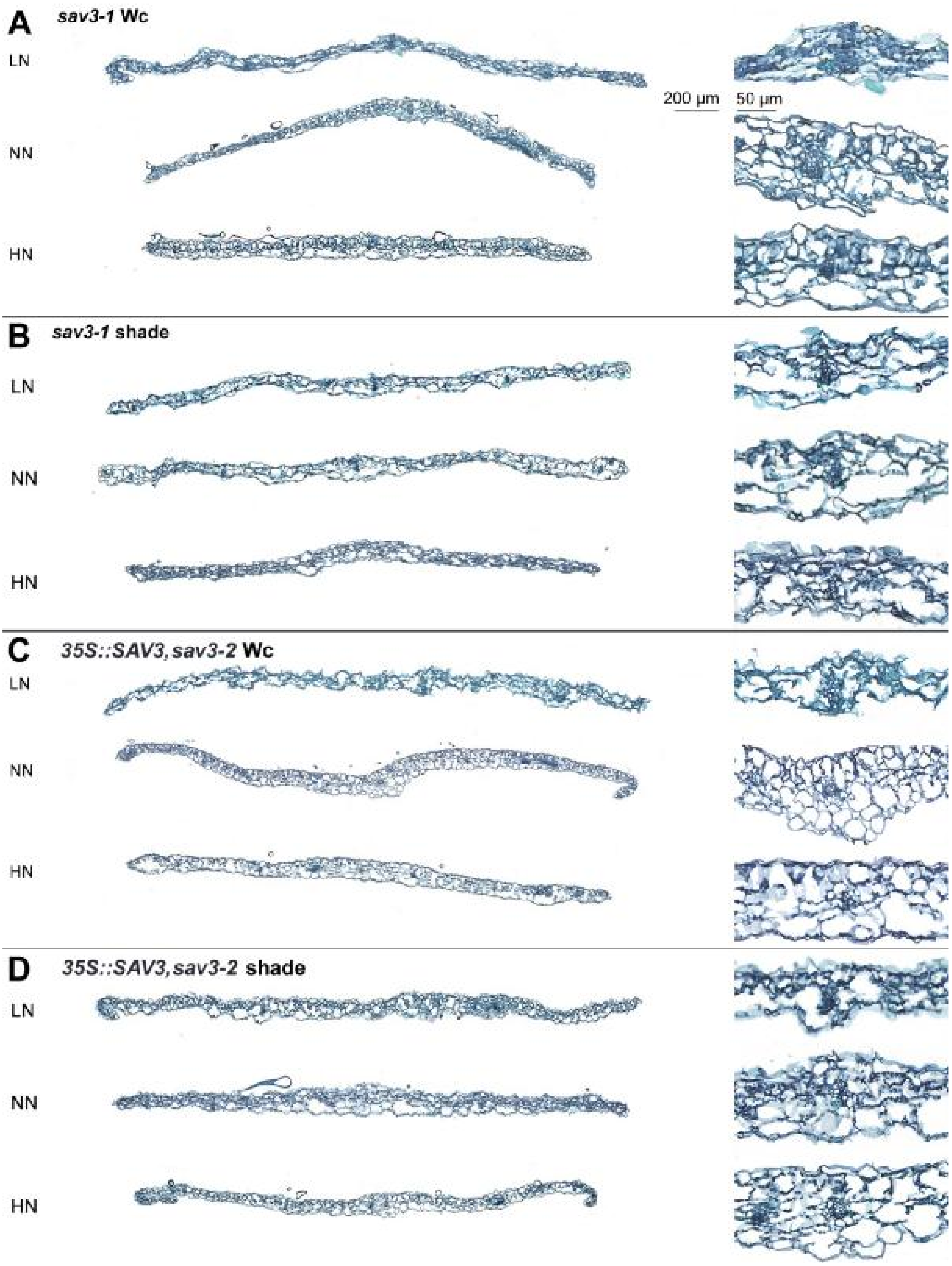

FIGURE S2 | *sav3-1* and *35S::SAV3,sav3-2* showed weak responses to N levels and shade treatment. (A–D) Cross sections of *sav3-1* and *35S::SAV3,sav3-2* leaves grown under different N levels and continuous white light (Wc) (A and C) or shade condition (B and D). Local enlarged images of the leaf central vein are shown in the corresponding right panels. LN, low N condition; NN, normal N condition; HN, high N condition.

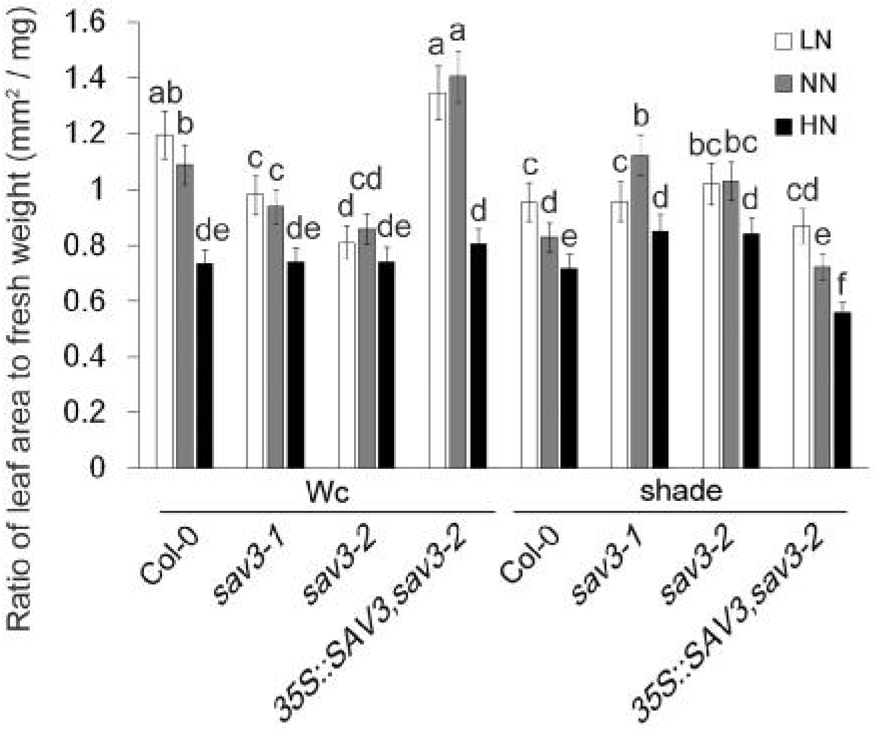

FIGURE S3 | Leaf area per fresh weight decreased under high-N treatment and shade treatment. Ratios of leaf area to fresh weight of 14-day-old leaves under different N levels with or without shade treatment were determined. LN, low N condition; NN, normal N condition; HN, high N condition. Bars represent standard deviations of 15 independent replicates. Values followed by different letters are significantly different at $P < 0.05$ according to Duncan's multiple range test.

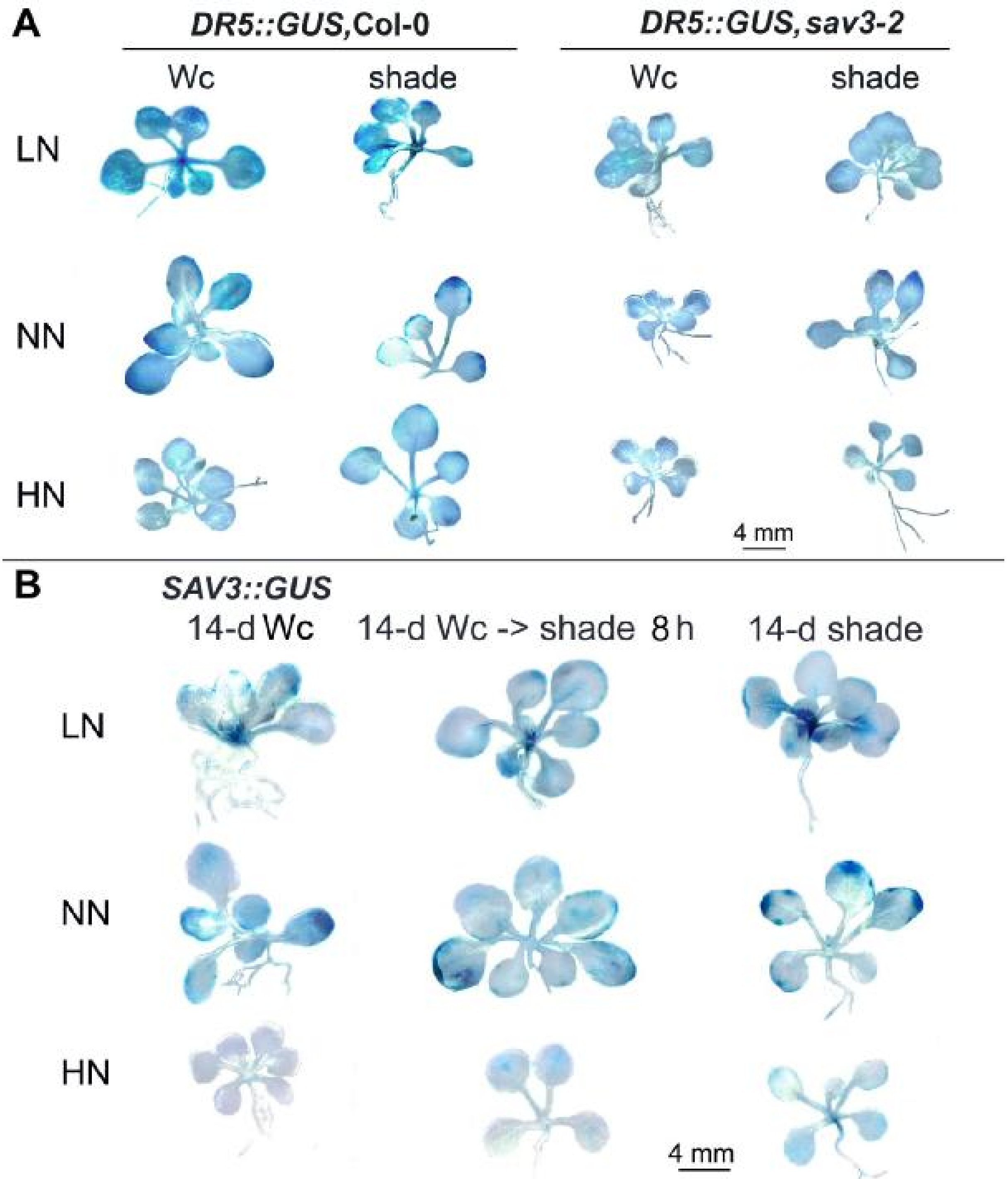

FIGURE S4 | Auxin in mature seedlings under shade conditions was reduced by high-N treatment. (A) Auxin accumulation pattern in 14-day-old wild-type (Col-0) and *sav3-2* mutant seedlings under different N levels with or without shade treatment. The levels of auxin were reflected by DR5::GUS expression. (B) *SAV3* expression pattern in 14-day-old wild-type seedlings under different N levels with or without (8-h or 14-d) shade treatment. The levels of *SAV3* gene expression were reflected by SAV3::GUS expression. Wc, continuous white light; LN, low N condition; NN, normal N condition; HN, high N condition.
